# Molecular and Clinical Prognostic Biomarkers of COVID-19 Severity and Persistence

**DOI:** 10.3390/pathogens11030311

**Published:** 2022-03-02

**Authors:** Gethsimani Papadopoulou, Eleni Manoloudi, Nikolena Repousi, Lemonia Skoura, Tara Hurst, Timokratis Karamitros

**Affiliations:** 1 Bioinformatics and Applied Genomics Unit, Department of Microbiology, Hellenic Pasteur Institute, 115 21 Athens, Greece; gesthpap@pasteur.gr (G.P.); elman12009@gmail.com (E.M.); nikolenarepousi@gmail.com (N.R.); 2 Department of Microbiology, AHEPA University Hospital, Medical School, Aristotle University of Thessaloniki, 546 36 Thessaloniki, Greece; mollyskoura@gmail.com; 3 School of Health Sciences, Birmingham City University, Birmingham B15 3TN, UK; tara.hurst@bcu.ac.uk

**Keywords:** SARS-CoV-2, COVID-19, pandemic, host immune response, prognostic biomarker, COVID-19 severity, COVID-19 persistence, transcriptomic signature, molecular biomarker, cellular biomarker

## Abstract

The coronavirus disease 2019 (COVID-19), caused by the severe acute respiratory syndrome coronavirus-2 (SARS-CoV-2), poses several challenges to clinicians, due to its unpredictable clinical course. The identification of laboratory biomarkers, specific cellular, and molecular mediators of immune response could contribute to the prognosis and management of COVID-19 patients. Of utmost importance is also the detection of differentially expressed genes, which can serve as transcriptomic signatures, providing information valuable to stratify patients into groups, based on the severity of the disease. The role of biomarkers such as IL-6, procalcitonin, neutrophil–lymphocyte ratio, white blood cell counts, etc. has already been highlighted in recently published studies; however, there is a notable amount of new evidence that has not been summarized yet, especially regarding transcriptomic signatures. Hence, in this review, we assess the latest cellular and molecular data and determine the significance of abnormalities in potential biomarkers for COVID-19 severity and persistence. Furthermore, we applied Gene Ontology (GO) enrichment analysis using the genes reported as differentially expressed in the literature in order to investigate which biological pathways are significantly enriched. The analysis revealed a number of processes, such as inflammatory response, and monocyte and neutrophil chemotaxis, which occur as part of the complex immune response to SARS-CoV-2.

## 1. Introduction

The COVID-19 pandemic poses a global health threat as it is responsible for more than 426 million cases of infection and 5.89 million deaths since December 2019 (https://covid19.who.int/, accessed on 1 February 2022), while it raises challenges in clinical assessment of the disease severity. It was initially considered to be a respiratory infection; however, based on the clinical experience on COVID-19 acquired during the past 18 months, SARS-CoV-2 infection is now recognized as a multisystem disease, resulting in a wide range of clinical manifestations [1,2]. The majority of COVID-19 patients (~80%) are asymptomatic or have symptoms such as fever, dry cough, and fatigue, which are characteristic of mild or moderate pneumonia [3]. Approximately 15% of cases develop severe disease with viral pneumonia. These patients exhibit dyspnea, arterial oxygen saturation (SpO_2_) ≤ 93%, respiratory rate ≥ 30 beats/min, partial pressure of oxygen (PaO_2_)/fraction of inspired oxygen (FiO_2_) < 300, lung infiltrates > 50% and as a result, their hospitalization is inevitable. Only around 5% of cases progress to critical illness, presenting acute respiratory distress syndrome (ARDS), shock or multiple organ failure (MOF), and require mechanical ventilation or admission to intensive care unit (ICU); approximately 2% of cases are ultimately fatal [4,5]. Risk factors, including age, and comorbidities such as obesity, hypertension, cardiovascular disease, type 2 diabetes, chronic pulmonary disease or malignancy [4,6,7], affect the course of the infection and the presentation of clinical manifestations, increasing the possibility of progression to severe disease, which can lead to persistence or even mortality. Recent studies have shown that elderly patients experience more severe clinical manifestations and increased risk of mortality compared to younger individuals, who are less vulnerable to severe disease [4,6,7,8,9,10]. Nonetheless, they are considered to be important contributors in the formation of molecular transmission clusters, groups of individuals who are associated with each other by SARS-CoV-2 transmission, thus representing a risk network [11].

Regarding persistent COVID—also known as long COVID, post COVID syndrome, post-acute sequelae of COVID-19 (PASC), chronic COVID syndrome (CCS), and long-haul COVID—the data are scarce. The condition is characterized by long-term sequelae persisting after the typical convalescence period of COVID-19. Greenhalgh and colleagues further subdivide the syndrome into post-acute and chronic, if the symptoms persist for three or twelve weeks, respectively [12]. An estimated 10% of COVID-19 survivors are considered as long-haulers and experience ongoing symptoms such as cough, dyspnea, chest pain, headache, difficulty concentrating, arthralgia, muscle pains, fatigue, and olfactory dysfunction for an extended period of time [12,13]. Experiencing such a debilitating disease in its severe form can result to prolonged recovery. While there are a number of risk factors linked to susceptibility to post-COVID syndrome, including age and comorbidities, it is noteworthy that there are cases in younger individuals and with no pre-existing medical conditions, who suffered from mild disease and yet developed long COVID [12,13].

It has therefore become clear that heterogeneity of COVID-19 symptoms and factors affecting the clinical outcome pose difficulties in the stratification of patients and prediction of the course of the disease. The intra- and inter-patient variability contribute to this, affecting the efficacy of the immune response [14]. The association of abnormalities in laboratory parameters and levels of prognostic and diagnostic biomarkers with disease outcomes through research of viral pathogens is valuable as it enables identification of pathogenic agents, successful clinical assessment, and prediction of therapeutic response [15]. The case of COVID-19 is not an exception, as the correlation of biomarker levels with the clinical outcome might be instrumental in clinical decision-making [16]. There are many different classes of biomarkers, mostly found in the human serum, including well-studied biochemical, hematological, and immunological parameters. For example, increased CRP concentration indicates severe COVID-19 infection, as its levels are significantly elevated during the initial stage of the disease in both cases of severe and critical patients, making CRP a perfect candidate as an inflammatory biomarker [17]. Moreover, prolonged activated partial thromboplastin time (APTT) and high D-dimer and/or fibrin degradation product (FDP) levels are considered to be signs of consumptive coagulopathy, and particularly, significant increase of the last two fibrin-associated markers is reported in all COVID-19 deaths [17]. In addition, according to a meta-analysis of 75 studies with 17,052 hospitalized patients 18 years of age or older, the comparison between severe/non-survivors vs. non-severe/survivors and biomarkers of inflammation and/or thrombosis pointed out that except for D-dimer, fibrinogen and CRP, the following thromboinflammatory biomarkers—high-sensitivity CRP, ferritin, interleukin, and high-sensitivity troponin—also serve as markers of end-organ damage and are correlated with increased severity and mortality in COVID-19 patients [18]. Yao et al. conducted a study that included 590 hospitalized COVID-19 patients, building a model to predict the risk of developing severe COVID-19 disease, with multivariate logistic regression. The result of the study is in agreement with the aforementioned, revealing that LDH, CRP, and ESR can be used as independent blood biomarkers, deployed along with age and comorbidities in order to establish a risk score [19]. Of increasing interest are the less well-known biomarkers including transcriptomic profiles [17,20] and gene signatures, which characterize the response to the virus and are crucial for disease prognosis. Immunological markers, including aberrant immune cell counts and proinflammatory cytokine levels, enable discrimination of disease severity; for example, lymphopenia is associated with severity as it is observed in 80% of individuals being critically ill [17,21]. Patients requiring admission to the ICU present a drastic decrease in CD8+ T cells, while in the elderly suffering severe disease, lymphopenia is frequently observed, and could be possibly associated with a higher mortality rate in this age group [22]. A cytokine storm characterized by elevation in the serum concentrations of several pro-inflammatory cytokines is a hallmark of COVID-19, with IL-6 and IL-10 being significantly elevated in those with critical disease [23]. Thus, measures of immune function, particularly lymphocyte counts and cytokine concentrations, could be used as biomarkers to predict severity and persistence of the disease [16,24].

**Figure 1:**
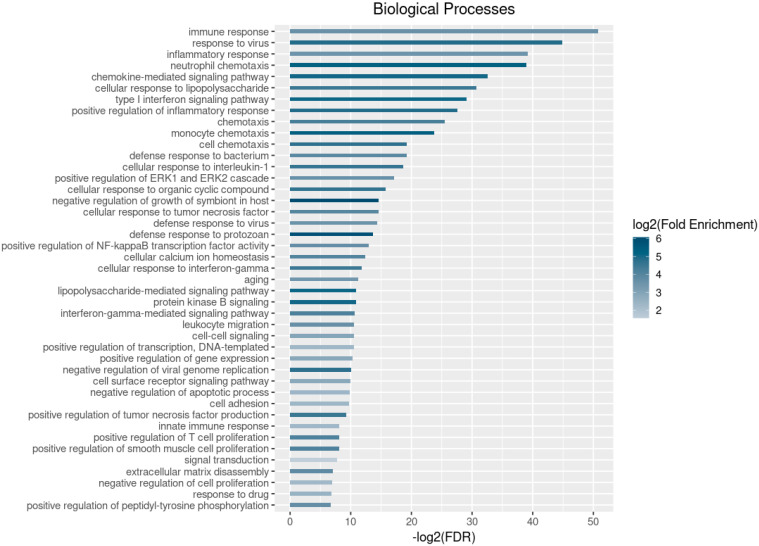
Significantly enriched biological processes (log2 fold of enrichment > 1.5 and FDR < 0.01) based on the Gene Ontology (GO) enrichment analysis of the differentially expressed genes between COVID-19 patients with severe/critical or mild/moderate disease.

Taking into account the valuable role of biomarkers in COVID-19 diagnosis and risk assessment, we performed this study, which has a twofold aim. First, we set out to describe serum and transcriptomic biomarkers by reviewing current research. For this purpose, we performed literature search using the PubMed/Medline and EMBASE databases. The identification of any remaining relevant published studies was performed using citation tracking. During the search, no date restrictions were applied and the language of selection was English. Data was extracted only from full-text articles. The studies considered as eligible for inclusion examined the association between severe or long COVID-19 and hematologic parameters, cell populations, viral load, SARS-CoV-2 antibodies, and transcriptomic signatures. Due to the large number of studies conducted to gain insight into the association between COVID-19 severity and alterations of hematologic parameters (including inflammatory markers, coagulation parameters, as well as circulating cytokines), we limited our research to those published between March 2020 and June 2021. The search strategy involved combining keywords for COVID-19 (“severe COVID-19”, “severe SARS-CoV-2 infection”, “long COVID-19”, “persistent COVID-19”), immune response (“immune response”, “host response”) and the biomarkers examined (“blood biomarkers”, “hematologic parameters”, “viral load”, “cell populations”, “transcriptome”, “transcriptomic signatures”). Due to the rapid advances and massive impact of COVID-19 on global public health, the literature concerning the viral infection was daily updated and the three authors independently reviewed the incorporated 105 studies based on the aforementioned criteria. Secondly, we further evaluated the role of differentially expressed genes in biological processes by performing gene ontology (GO) enrichment analysis using the bioinformatics resource DAVID (v 6.8). The gene set analyzed consists of the genes that were found to be up-regulated and down-regulated. The top 43 significantly enriched GO categories (FDR < 0.01, log2 FC > 1.5) are shown in Figure 1.

## 2. Association of Common Blood-Based Biomarkers and COVID-19 Severity

A number of blood inflammatory and coagulation biomarkers are linked to the severity of COVID-19 infection. Specific alterations in cellular populations such as lymphopenia, high neutrophil count (3.74 × 10^9^/L) and neutrophil-to-lymphocyte ratio (NLR), higher levels of white blood cells (WBCs), and thrombocytopenia are associated with severe disease and fatal outcome (Table 1) [16,17]. Elevated levels of inflammatory markers (e.g., CRP and ferritin), liver enzymes, lactate dehydrogenase (LDH), creatine phosphokinase (CPK), D-dimer (>1 mcg/mL), prothrombin time (PT), troponin, as well as high levels of inflammatory cytokines (IL-6, TNF-α) have been associated with worse clinical outcome [17,25]. 

A meta-analysis of 6320 patients reported that patients having elevated levels of IL-6, IL-10, ESR, and PCT were more prone to develop severe-stage COVID-19, while high levels of D-dimer, PT, and rapidly lowered levels of fibrinogen at the latter stages of hospitalization were specifically observed in non-survivors [16,26]. Moreover, Huang et al. confirmed in their meta-analysis the association of LDH, CRP, and PCT with the severity of the infection and additionally, demonstrated that levels of leukocytosis, lymphocytopenia and aspartate aminotransferase (AST) are linked to different degrees of risk for COVID-19 severity [27]. Consequently, this study showed evidence that serial WBC count, lymphocyte count, CRP, procalcitonin (PCT), LDH and AST measurements could be used as biomarkers for severe COVID-19 [27]. This is also supported by the study of Ou et al. who observed this trend, including elevation of alanine transaminase (ALT) in non-survivors compared to those who survived, although the two groups had approximately the same levels of platelet counts and LDH [28]. The meta-analysis of Feng and colleagues [29] confirmed previously mentioned findings about the levels of PCT, CRP, erythrocyte sedimentation rate (ESR) and IL-6, as well as the fact that increased WBC, decreased numbers of total lymphocytes and subtypes, such as CD4+T and CD8+T, and increased NLR are considered as risk factors for disease progression in patients with COVID-19. The significance of NLR as a biomarker is confirmed by another meta-analysis that included 44 studies and 7865 patients [30] and pointed out that NLR as well as a number of parameters, such as ferritin, PCT, serum amyloid A (SAA), IL-2, IL-2R, IL-4, IL-6, IL-8, IL-10, TNF-α and INF-γ, were significantly increased in the severe group compared to the non-severe group. 

Original research about long COVID-19 is still limited. According to a cross-sectional study, 30.1% and 9.5% of patients discharged with elevated biomarkers had consistently high levels of D-dimer and CRP, respectively [31]. In another study that examined the inflammatory response after SARS-CoV-2 infection, by comparing 10 healthy controls and 10 patients 40–60 days post-viral infection, a significant ongoing inflammatory response as well as mitochondrial stress in all patients was observed [32]. Mitochondrial stress is caused by insults that have as a result the loss of mitochondrial membrane integrity and membrane potential, metabolic dysfunction, alterations in energy intermediates, and impairment of mitochondrial translation. It is especially characterized by impaired OXPHOS and increased levels of mitochondrial ROS (mtROS) [33]. Specifically, among proteins with significantly altered levels was the N-Myc downstream regulated gene 1 (*NDRG1*), which, when deficient, affects macrophage differentiation and mast cell maturation. Moreover, elevated levels of the anti-inflammatory molecule collagen triple helix repeat containing 1 (CTHRC1) indicate that tissue damage has occurred even in moderately-ill patients, as this protein acts as M2 macrophage recruiter and regulator of Notch and TGF-β pathways, thereby promoting wound healing [32]. Elevated levels of IL-10 in severe patients negatively regulates cystatin C, while mild patients have higher levels of this protease inhibitor, used as a prognostic biomarker [32]. Levels of LDH higher than 750 U/L, together with other factors such as obesity and smoking status, can strongly predict risk of persistent abnormalities on chest X-rays (CXR) [34]. They also found elevated levels of a number of serum molecules like D-dimer, PCT, CRP, and albumin in severe patients. However, the study of Hariyanto et al. associated lower albumin levels (cutoff value 38.85 g/L) with poorer prognosis in COVID-19 patients [35]. The same study highlighted that the most powerful biomarker for early recognition of lung injury in severe COVID-19 is LDH (cutoff value 263.5 U/L).

## 3. Association of Alterations of Cell Populations and COVID-19 Severity

A number of studies have reported changes in immune cell populations that discriminate mild from severe patients upon admission to hospital and that could serve as early predictive biomarkers. Regarding cells considered to be mainly part of the innate immune system, neutrophils consist the first line of defense against pathogens, being the central players of innate response [36], while they also drive various aspects of adaptive immunity [37]. The contribution of neutrophils to host defense against viral respiratory infections is diverse and complex and this review attempts to provide updated information on the implication of neutrophils in mild compared to severe disease. According to several studies, the proportions of neutrophils were increased in severe patients compared to mild [38,39,40,41]. In particular, severe hospitalized patients had higher levels of CD16^low^, CD16^Int^ compared to mild and moderate ones, while increased levels of CD16^Int^ associated with elevated levels of D-dimer are reported in a severe patient until he passed away [39,42] and mature and activated ICAM-1 neutrophils [43]. The latter likely boost the proinflammatory cascade in COVID-19, as they contribute to inflammation spreading through reverse transendothelial migration, neutrophil aggregation, and effector functions [44,45]. Neutrophilia has been suggested as a predictive marker of disease severity [46]; however, according to Rebillard and colleagues, elevated neutrophil counts are predictive of disease severity in general and not COVID-19 specifically [43]. In general, the studies referring to granulocytes, including neutrophils, eosinophils, and basophils, present contradictory results. While one study found significantly reduced granulocytes in severe patients compared to mild [47], another described increased immature granulocytes in severely ill patients [39].

Characteristic of severe patients is the increased abundance of classical or immature monocytes (CD14+ or CD14++CD16−) [40,48,49,50]. Higher proportions of CD1aCD14+ and of monocytes expressing the cell adhesion molecule ALCAM, implicated in leukocyte transendothelial migration [51], were observed in patients who had an adverse outcome after 30 days [43]. CD14+ monocytes express CD1a, after being exposed to IL-4 and GM-CSF, and CD1a is involved in the presentation of lipid antigens to T cells [52]. Nevertheless, nonclassical (CD14−CD16++) and intermediate or transitional (CD14+CD16+) monocytes were significantly reduced in severe patients [39,48,49,53,54,55], while the vast influx of monocytes and neutrophils has been correlated with heightened inflammatory response, also triggering ARDS [40]. After critically reviewing the aforementioned, the reduction in nonclassical monocytes is proposed as a predictive marker of severity in COVID-19. In severe COVID-19, dendritic cells (DCs) and a specific subset, CD1c+ conventional dendritic cells (cDCs), significantly decreased, implying a link with poor prognosis [49,50,54,56]. These antigen-presenting cells can induce CD4+ and CD8+ T cell responses and as a result, they are quite important for effective adaptive immune responses against viral infections [57]. In addition, Laing and colleagues reported depletions of plasmacytoid dendritic cells (pDCs) in severe COVID-19 [58]. pDCs link innate and adaptive immunity and one of their key functions is the production of IFN-I in response to viral or bacterial infections [59]. Depletion of pDCs could therefore result in a reduced ability to detect and limit viraemia since high viral loads have been associated with severe disease [60]. Changes in the composition of adaptive immune cells are associated with disease progression and it is reported that severe and critical COVID-19 patients present dramatically decreased natural killer cells (NK cells) on admission to hospital [43,49,53]. Despite the general decrease in NK cell population, a significant increase in the subset of the activated NKs was observed [40]. More specifically, cytopenia of the CD3-CD56^bright^CD16^dim^ NK cell population is correlated with severe disease progression [39]. CD56^bright^ NK cells are responsible for mass cytokine production, while CD56^bright^CD16^dim^ cells are considered to be an intermediate stage between the most immature (CD56^bright^CD16−) and the most mature (CD56^dim^CD16+) cell types. CD56^bright^CD16^dim^ NK cells exhibit weak antibody-dependent cellular cytotoxicity [61]. Despite the general decrease in NK cell population, a significant increase in the subset of the activated NKs was observed [40].

In general, elevated proportions of B cells together with changes indicative of a proinflammatory B cell profile [43] are observed in severe [40,50] and critical [48] COVID-19 patients. Several different research groups found selective expansion of B-cell plasmablasts (PBs)—short-lived cells that derive from memory B cells—in severe COVID-19 [56,62]. This suggests that these patients exhibit a strong specific SARS-CoV-2 antibody response [63] since plasmablasts differentiate into antibody-secreting plasma cells. However, this trend is not observed in all subpopulations, as CD5+, peripheral naïve, and memory B cells were markedly reduced in individuals with severe COVID-19 or those who had serious complications after 30 days [43,53,62,63]. Although peripheral naïve and memory B cells, also known as B-1 lymphocytes, traditionally belong to the adaptive immunity, they have some features of innate immune cells too [64]. This subpopulation plays an important role in immune response since they produce low-affinity polyreactive antibodies [65] and IL-10 [66].

Decreased frequencies of T cells, especially naïve CD4+ and CD8+ T cells, were observed in individuals with severe or critical disease [39,40,49,50,53,67,68,69]. A similar decrease is characteristic of long COVID-19 patients who presented lower CD4+ T cell counts, compared to other groups of patients [70]. Regarding the non-exhausted CD8+ T cells expressing low levels of the immune checkpoint proteins PD-1, CTLA-4, and TIGIT, their population is significantly reduced in the severe sub-cohort and this depletion may lead to impaired cell mediated immune response to SARS-CoV-2 [47]. A decline in the number of regulatory T cells (TReg), as well as in their suppressive activity, was also observed in severe COVID-19 patients [71]. Of note is that at the same time, an increase in terminal effector and effector memory CD8 T cells were observed in the severe condition [69], a finding in agreement with the increased activation of memory CD4+ and CD8+ T cells reported in these patients [56]. Other studies also refer to the presence of CD38, HLA-DR, Ki-67, and PD-1 memory CD8 T cells in acute moderate or severe patients [56,72]. SARS-CoV-2-specific CD4+ T cell responses were also frequently observed in unexposed individuals, suggesting the possibility of pre-existing cross-reactive immune memory to seasonal coronaviruses [73]. According to an interesting observation supporting the hypothesis of reactivation of existing memory CD8 T cells, patients who recovered from SARS possess long-lasting memory T cells that are reactive to the N protein of SARS-CoV 17 years after the outbreak of SARS in 2003; these T cells displayed robust cross-reactivity to the N protein of SARS-CoV-2 [74]. In addition, individuals with acute infection had increased polarized Th1 and Th2 cells [68], which are the coordinators of cell-mediated and humoral response, respectively, whereas Huang and colleagues found lower proportions of Th1/Th17 cells in critical condition [69]. An adequate Th1 response can eliminate the viral infection, once established. Otherwise, an exacerbation of the disease leading to a cytokine storm seems inevitable, with Th2 cells that are linked to poor prognosis taking control [75]. The same trend was described for γδ T cells, which were also decreased in severely ill patients [50,53]. This type of cells exhibits a broad antiviral activity and their potential role in therapies against COVID-19 is being investigated [76]. Severe and critical COVID-19 patients presented elevated levels of megakaryocytes (MKs), hematologic progenitors of platelets, leading along with other factors to hypercoagulation [50,62,68]. This elevation could also exacerbate the cytokine storm observed in severe COVID-19 cases, as MKs, together with monocytes and specific T cell subtypes, may contribute to this phenomenon [50]. In addition, MKs exhibit antiviral activities as has been shown in cases of DENV infection [77].

## 4. Association of Alterations of Anti- SARS-CoV-2 Antibody Levels and COVID-19 Severity

IgG and neutralizing antibodies (nAbs) were identified more frequently in severe COVID-19 patients at baseline [43], with nAbs being identified in 100% of severely ill patients, compared to the 95% in mild [78]. Similarly, increased levels of specific anti-SARS-CoV-2 IgG antibodies are detected in critically ill patients compared to other groups (70% in ICU, 52% in the outpatients, 49% in ward groups, *p* < 0.05); however, it should be noted that the samples from patients with anti-SARS-CoV-2 IgG presence were collected later after disease onset contrary to those with absence of IgG (10.0 days; 7.0 days, *p* = 0.003) [6]. Interestingly, Wang and colleagues underline the fact that the early production of antibodies does not necessarily lead to early elimination of SARS-CoV-2; however, the titer and specificity of antibodies may play a more important role towards virus eradication [79]. As far as long COVID is concerned, this syndrome may be favored by low levels of neutralizing antibodies [79].

## 5. Association of Alterations of SARS-CoV-2 Viral Load and COVID-19 Severity

In viral infections, including HIV, Ebola, and influenza, viral titers are considered to be a predictor of disease severity and progression [80]. COVID-19 is not an exception, as knowledge of viral load and its impact on disease severity and outcome is of high importance in order to develop strategies of antiviral treatment and restrain the infection. In the plasma of critically ill patients who do not survive, higher viral RNA loads are reported compared to the other patient groups (*p* < 0.001), which indicates that viral load is strongly associated with the severity of the disease [6]. More specifically, the viral load in ICU non-survivors was 1587 copies/mL (N1 region) and 2798 copies/mL (N2 region), whereas the viral load in ICU survivors was 574 copies/mL (N1 region) and 523 copies/mL (N2 region), and in outpatients and ward patients, the corresponding values were 0 (*p* < 0.001) [6]. The former observation is supported by the findings of Ren et al., who found that the genes encoding regions of IgA1, IgA2, IgG1, and IgG2 in plasma B cells were upregulated in severe patients [50]. Further, no significant diversity in the initial viral titer was reported between asymptomatic patients or those exhibiting mild disease and patients with severe disease requiring respiratory support during hospitalization; however, after the establishment of the inflammation in patients who received supplemental oxygen and deceased in hospital, the viral titer remained high, suggesting that viral load might be related to mortality [53]. Other studies have also found that the SARS-CoV-2 viral load is independently correlated with and predictive of mortality [81,82]. On the contrary, long-term SARS-CoV-2 infection in patients exhibiting post initial symptoms for more than 50 days was characterized by low viral load accompanied by low viral pathogenicity [79].

## 6. Association of Alterations of Transcriptomic Signatures in COVID-19

**Figure 2:**
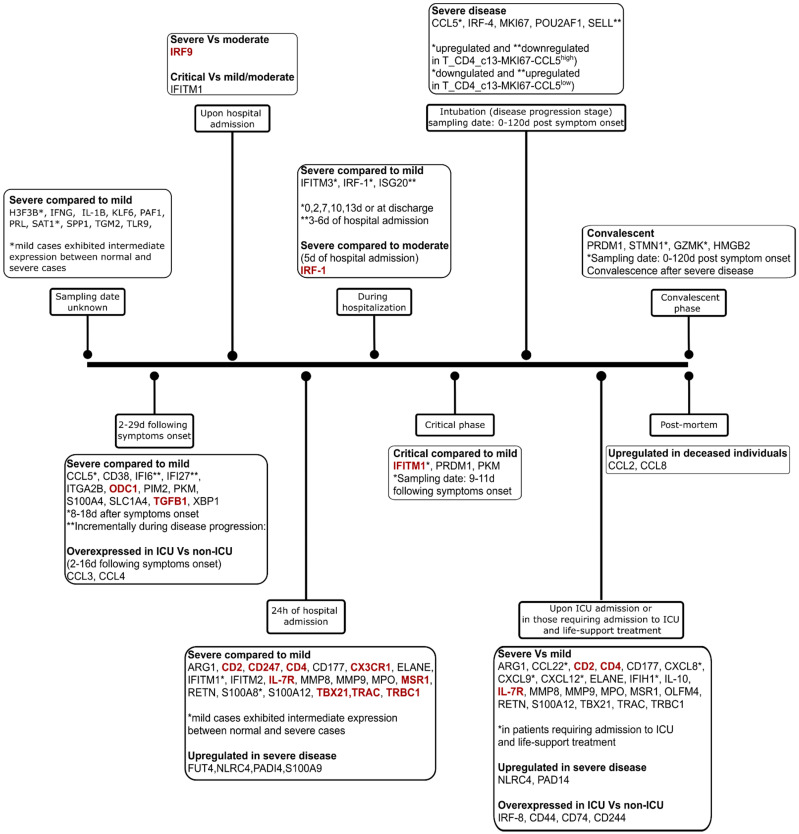
Timeline presenting the genes that are differentially expressed in COVID-19 patients in different severity stages and in various time points during the infection. A small number of genes (colored red) is downregulated, while the majority of the genes (colored black) is upregulated.

During our detailed COVID-19 literature research, we mined information regarding genes and proteins that received significant attention due to their involvement in severity and progression of COVID-19. The genes differentially expressed in patients in different stages of SARS-CoV-2 infection, as well as the representative gene symbol, the corresponding Entrez ID number, and the aliases are enlisted in Supplementary Table S1. We were particularly interested in the role of these differentially expressed molecules in various phenotypes ranging from mild disease to ARDS [83]. The great majority of the genes that alter their expression during host immune response was found to be upregulated, while only 13 genes were downregulated, and the alteration was observed mainly in patients between severe and mild condition (Figure 2). It should be noted that the *IFITM1* gene was found to be overexpressed upon hospital admission and downregulated when the sampling date was 9–11 days following symptoms’ onset, in patients who suffered critical compared to mild or moderate disease. Findings of other studies also support that the gene expression is altered in a robust way in blood samples of patients suffering from severe COVID-19 compared to mild/moderate, as a great number of genes are differentially expressed and are involved in a variety of immune response pathways [38]. For example, TNFα signaling and inflammatory pathways enriched in severe patients are indicative of the innate immune response activation [84], while on the other hand, in mild patients, pathways linked to IFN-α/γ responses are significantly enriched [84].

### 6.1. Gene Expression Alterations in Different Immune Cell Populations and COVID-19 Severity

Differentially expressed genes in individuals suffering severe disease are probably associated with activation of neutrophils and granulocytes. Aschenbrenner et al. specifically report that *ELANE*, *OLFM4*, *MPO*, *RETN*, *ARG1*, as well as the genes *CD177* (associated with granulocyte activation) and *S100A12* (upregulated in granulocytes), are highly expressed in severely ill patients requiring admission to the ICU rather than in mild cases (Figure 2) [38]. The study of the granulocyte transcriptome 24 h after hospital admission in patients suffering severe disease revealed upregulation of *CD15*, *S100A8/9*, *PADI4*, *NLRC4*, *MMP8*, and *MMP9* (Figure 1) [38,84] and overexpression of genes encoding matrix metallopeptidase 9/25 (MMP9/25) in neutrophils and macrophages of severe patients is associated with the promotion of leukocyte migration to inflamed sites [84]. There are a number of genes that are downregulated either exclusively in severe disease or incrementally as the disease severity progresses, including *CX3CR1* and *MSR1* related to monocytes and macrophages, respectively [38], HLA class II paralogs (*HLA-DQB1/2*, *HLA-DMB*, *HLA-DRA*, and *HLA-DPB1*) indicating impairment of the antigen-presentation process by APCs [84], as well as genes *TRAC*, *TRBC1*, *CD247*, *CD4*, *CD2*, *TBET*, and *IL7R* which are associated with lymphocytes [38], and *SKAP* and *LAG-3*, involved specifically in the activation of T cells [84]. HLA class II molecules were particularly downregulated in those in need of ventilation [85].

Multiple studies highlight elevated expression of a number of genes involved in B cell activation and differentiation to plasmablasts in patients with severe COVID-19. Parallel single cell RNA-seq (scRNAseq) and multiplex serum ELISA were performed on samples from 13 inpatients, a recovery control, and 14 healthy individuals, revealing that B cells were depleted, whereas proportions of PBs were elevated from the time of the infection until the early convalescent phase [62]. This elevation was associated with high levels of TNF, IL-10, and IL-21. Of note, the IL-21 cytokine expressed in T, B, and NK cells is involved in plasma cell differentiation of B cells by inducing *BLIMP-1* via STAT3 signaling [62,86]. *BLIMP*-1 encodes PRDM1, which appears to be significantly activated at critical and convalescent phases [62]. 

Increased PBs in severe versus mild disease, accompanied by the presence of IFN-I related gene products (e.g., *IFI27*, *IFI6*, *IFITM1*) characterize the inflammatory response in every phase of the disease except critical [62]. Other transcripts such as XBP1 (involved in ER stress and protein folding) and *PIM2* and *S100A4* (cell proliferation) remain elevated during the incremental phase of inflammation in patients with severe disease (sampling date 2–29 days following the symptoms onset (Figure 2) [62]. Zhu and colleagues [87] compared healthy donors with COVID-19 patients, observing that patients show upregulation in the representative genes *PRDM1*, *XBP1*, and *IRF4* involved in B cell activation pathways. Ren and colleagues specifically associated upregulation of the aforementioned genes, including *MKI67* (pointing out activation of proliferation) and *POU2AF1* in plasma cells, with disease severity [50]. *IRF4*, together with *BATF* and *CD27*, are genes associated with T cell exhaustion, and, the more severe the disease is, the higher their expression in the blood samples [40]. Two T-cell populations are elevated in severe patients: T_CD4_c13-MKI67-CCL5^low^ cells expressing high and low levels of *SELL* and *CCL5*, respectively (particularly in the disease progression phase), and T_CD8_c10-MKI67-GZMK cells with upregulated *STMN1*, *HMGB2*, *MKI67*, and *GZMK* mainly in the convalescent phase [50]. The increase in PBs in severe COVID-19 patients is also followed by overexpression of *CD38*, *SLC1A4* involved in response to unfolded proteins, and ATP synthesis in mitochondria [62,68]. Zhou et al. performed an analysis using PBMCs samples from COVID-19 patients in different aspects of the clinical spectrum in order to screen factors that could serve as predictors of disease severity by building a machine-learning model. The analysis revealed that severity was either inhibited by signals that have antiviral-activity (*CD44*, *IRF8*, and *CD244*) or are characterized as immune-negative (*TGFB1*, *TGFBR1*, *TGFBR2*, and CD74) or was promoted by signals that act as pro-inflammatory cytokines, like CCL3, CCL4, and the already mentioned IL-1 and TNF-α) [68]. It should be noted though that CCL3, CCL4, and other proinflammatory molecules, such as TNF-α, IL6, IL1B, and CXCL2, are not secreted by peripheral monocytes and leukocytes (T and NK cells); thus, these cells are not considered as responsible for the cytokine storm phenomenon [85]. Activation of regulators such as IFNG, PRL, TGM2, TLR9, PAF1, IL1B, TNF-α, and NFkB in BAL samples of severely-ill individuals should also be reported [88].

### 6.2. Association of Interferon Activity with COVID-19 Severity

According to the results of the GO enrichment analysis, a number of differentially expressed genes was related to the GO terms type I interferon and interferon-gamma-mediated signaling pathways, indicating that IFN-I and II responses are predominant in patients with severe COVID-19 (Figure 1). Indeed, pathways of innate immunity and inflammation are activated in a way that is disease severity-dependent and IFN-I and II response is more robust in moderate patients compared to mild, severe, critical, and cured group [40,69,84], not only driving a successive response to viral pathogens, but also preventing the establishment of a hyperinflammatory state due to neutrophil and monocyte hyperactivation [84]. Regarding IFN-II response, levels of IFN-γ are elevated mainly in mild to moderate patients and slightly increased in severely ill individuals, but not in those in critical condition [40]. Hadjadj et al. reported contradictory results, as they performed Simoa digital ELISA, revealing that the type I interferon IFN-α2 levels are significantly lower in critical patients, compared to those suffering mild or moderate disease, despite the fact that mRNA levels were elevated in severe patients; it should be taken into account, though, that mRNA levels slightly exceeded detection limit [40]. Reduced IFN-α2 secretion in peripheral WBCs could be explained, as the predominant producers of IFN-α2, pDCs migrate to infection sites during the course of disease [40]. At the peak of the disease, IFN-related mRNA levels are elevated [62], while in the severe and critical phase, transcripts like *IRF3*/*7*/*8* and *MAVS* are suppressed [84]. Probably due to the suppression of translation as a consequence of IFN-I response, levels of mRNAs encoding ribosomal structural proteins remain reduced until the late convalescent phase [62], a fact supported by the decrease in the number of naïve CD4+ T lymphocytes, which present elevated expression of ribosome coding genes, such as *RPL32*, *RPL30*, *RPS12*, and *RPS3A*, in hospitalized patients [69]. Overexpression of genes involved in IL-1β and vasodilatory signaling is reported during the incremental period between the early infection and critical phase [62]. Along with IL-β, other inflammatory mediators, such as IL-2, IL-4, and TNF-α were found highly expressed during the innate immune response of severely ill patients [89]. Contrary to what is mentioned above, even though IL-1β mRNA levels were elevated, the cytokine was not elevated in its active form, probably due to unsuccessful cleavage and secretion [40]. Blanco-Melo et. al. [90] performed a comparison of lung biopsies of infected individuals who passed away to lung tissue with healthy donors and made the note-worthy observation that even though no IFN-I or IFN-III were detected by RNA-seq or semi-quantitative PCR, the synthesis of ISG products involved in innate immunity and response through cytokine secretion was induced. Specifically, among the circulating molecules driving COVID-19 pathology, the chemokine CCL11 and the neutrophil, T-cell and NK chemoattractants CXCL8, CXCL9, and CXCL16, respectively, were found to be elevated [90]. Other overexpressed genes encode recruiters of monocytes and macrophages, *IL-6*, *IL-1RA*, *KLF6*, *SPP1*, *CCL3*, *CCL2*, and *CCL8* [90]; it should be noted that all but IL-6 and IL-1RA achieve significantly higher levels among severely ill individuals compared to mild (intermediate expression) and the uninfected. They can thus serve as gene signatures to reveal severely ill individuals [88]. These findings are in agreement with the results of the GO enrichment analysis we performed, as some of the most significantly overexpressed and down-regulated genes are involved in the neutrophil and monocyte chemotaxis (the terms chemotaxis and cell chemotaxis have also been found to be enriched), as well as in chemokine−mediated signaling pathway (Figure 2). Except for overexpression of CXCL9, Jain et al. observed high concentrations of CCL22 and CXCL12 in patients with severe, rather than mild or moderate disease [91]. Levels of CXCL2 produced by neutrophils implicated in inflammation are also elevated, indicating an increase in circulating proportion of this cell type, which may have prognostic value for the identification of patients prone to develop severe disease [90]. On the contrary, the levels of the receptor of CXCL2 (CXCR2) and not the levels of the chemokine itself are elevated in severe and critical disease [40]. In general, the involvement of pro-inflammatory molecules during the course of the disease was associated with increased severity and adverse clinical outcome [71]. 

IFN-I stimulated genes differentially expressed are *IFITM1*, *IFITM2*, *IFITM3*, *PKM*, and *ITGA2B* (*CD41*) that are overexpressed in megakaryocytes (MKs) of individuals suffering severe disease and *ODC1* and *TGFB1*, which are downregulated [62]. *IFITM1* overexpression in the plasma of severely ill is confirmed by Lee et al., who demonstrated that the expression of various ISGs, such as *IFITM2*, *IFITM3*, *ISG20* and genes activated as a response to TNF/IL-1β activation, is elevated [49]. Other studies also suggest that *IFITM1* and *IFITM2* serve as gene signatures for severe disease as, together with H3F3B, *SAT1*, and *S100A8* in neutrophils, they are highly expressed in severe patients compared to mild and uninfected [40,88]. The quantification of the expression of genes related to immune response in peripheral WBCs revealed that the expression of ISGs, such as *IFITM1*, *IFIT2*, and *MX1* is suppressed in critical patients, while *IFNAR1*, *JAK1*, and *TYK2* (the products of which are involved in IFN-I signaling) are upregulated [40]. Of note, in case of other viral infections such as measles, the RNA-seq analysis of the pharyngeal epithelium of patients revealed that IFIT2 is upregulated and its protein product is associated with response to viral infection [92]. Other ISG products in high concentration in severely ill patients compared to mild or moderate ones are IFIH1, IFI44, IFIT1, and IL-10 [93]. Regarding the novel biomarker IFI27, it is known to reflect active influenza virus infection, as it appears to be co-expressed with many genes with anti-viral functions and its TLR7-driven expression is observed in DCs and NK cells [93]. In contrast to the above, others report that there is no difference in the expression of *IFI27* and *IFITM3* between severe and mild samples [38] and a study that is currently under peer review supports that the alterations in gene expression reflect only local activity of IFI27 in the lower respiratory tract, as in the lung tissue of deceased individuals suffering COVID-19, *IFI27* was upregulated and the overexpression is correlated with high viral load [94]. On the other hand, a significant association between *IFI27* levels in the upper respiratory tract (nasopharyngeal IFI27) and the severity of the disease was reported [94]. This IFN-I related gene is strongly upregulated in COVID-19 patients too and in fact is more elevated in those with severe disease, compared to the mid- and moderate ones, whereas its levels are far lower in critical patients [69]. Data derived from RNA-seq experiments using lung tissues from deceased individuals revealed that there are genes differentially expressed in monocytes of severe patients, including *ISG15* and *JAK3* which are elevated, and the downregulated *RPS18* [49]. In general, response to IFN signaling by B, NK, and T cells is more robust in individuals from the severe group who also have higher viral titers and prolonged viral clearance [95].

The transcriptomic and KEGG pathway analysis of PBMCs of COVID-19 patients revealed that the main causes of lymphopenia might be the activation of apoptotic and P53 signaling pathways. Specifically, in the surface of T- and B cells, as well as in monocytes, in severe patients compared to the other groups, elevated expression of apoptotic signals is reported. Along with the suppression of expansion pathways, resulting in lower proliferating rates, these signals could be driving the lymphopenia [95,96]. This finding is supported by in situ experiments using TUNEL staining, which present that there is high rate of apoptosis in the spleens and lymph nodes in non-survivors, accompanied by overexpression of the death receptor FAS [97]. Moreover, in patients with severe and critical disease, T cells express inhibitory molecules, markers of exhaustion, such as T cell immunoglobulin and mucin-domain containing-3 (Tim-3) and programmed cell death protein 1 (PD-1) [95]. 

### 6.3. The Establishment of Hypoxia

A condition of hypoxia is being established during different time points of the disease, each time accompanied by a different subset of genes with altered expression. Elevated levels of transcription factors, such as IRF1, STAT3, and the hypoxic signal HIF1A (hypoxia-inducible factor-1a), involved in inflammation and IFN signaling are also reported during active disease [62]. Opposite to that, it has been reported that *IRF1* and *IRF9* expression is lower in severe patients compared to moderately ill, resulting in a deficient response of monocytes in IFN(-I) signaling, while on the other hand, elevated *IRF7* expression in lymphocytes results in a more robust IFN-I response [95]. In neutrophils and MKs of critically ill patients, a strong increase in PKM transcript counts (*PKM2*) was observed [62,98]. The pyruvate kinase PKM2 encoded, forms dimers, and interacts with the transcription factor HIF1A, promoting its activity in hypoxia and inflammation response, and especially its role in transcription of cytokines IL-1β and IL-6 [98]. 

### 6.4. Association of Alterations of Inflammatory Cytokines Levels and COVID-19 Severity

The inflammatory response is the third biological process found to be enriched in the GO enrichment, while a large number of significantly differentially expressed genes are involved in the positive regulation of inflammatory response, cellular response to IL−1, and cellular response to tumor necrosis factor (Figure 1). Overexpression of pro-inflammatory cytokines IL-1β and IL-6, along with downregulation of anti-inflammatory IL-10 is known to be involved in airway inflammation in ARDS, while increase in the ratio IL-6:IL-10 and IL-1β:IL-10 is also observed in severe clinical presentation compared to mild and moderate [98]. Overproduction of IL-6 was observed in deceased individuals over survivors and in those suffering severe over mild disease [99], and particularly in those hospitalized, IL-6 remained high for a long period of time [87]. Yao et al. also report in patients experiencing severe disease, higher circulating levels of IL-6, as well as TNF-α and CXCL10 (C-X-C motif chemokine ligand 10), which remain high for weeks during the infection and this inability to achieve normal concentration for a long period result to decreased ability to terminate the infection [95]. It should be noted though that the increase in the levels of IL-6 protein and *IL6R*, *SOCS3*, and *STAT3* genes induced by IL-6 is not reflected at the transcriptional level, as IL-6- mRNA was not detected in the PBMCs [40]. The same discrepancy is observed in case of TNF-α, where the factor itself, as well as genes induced by it are upregulated, contrary to TNF-α mRNA levels which were moderately increased [40]. Elevated expression of TNF-α, IL-2R, IL-1, IL-6, IL-8, and IL-10 (previously reported as downregulated [98] in severe cases compared to non-severe/mild ones, is related to exacerbation of inflammation and damage of vascular cells of the endothelium when accompanied by the accumulation of peroxides [68,71]. The negative regulation of endothelium repair is also supported by downregulation of CD47/SIRPA, CD24/SIGLEC10, and SLAMF7 signals, which results in the release of proinflammatory cytokines towards local and remote tissues causing systemic injury, exacerbating the damage induced by activated macrophages to endothelial cells in ICU-admitted patients [68]. Contrary to what is reported by McElvaney et al. about anti-inflammatory cytokine IL-10, secreted by activated Th2 cells [16], other studies report that its levels are higher in the plasma of COVID-19 ICU patients compared to non-ICU [88,100], and specifically higher in deceased compared to those recovered [101]. Additionally, Hadjadj et al. claim that both mRNA and protein levels of IL-10 are elevated in individuals suffering severe and critical disease [40]. The compensatory anti-inflammatory response syndrome (CARS) is responsible for the state of suppression of the immune system, part of the effort to restore homeostasis after inflammation, and the elevation of IL-10 levels might be associated with this state [99,102]. Other plasma cytokines with concentrations higher in patients admitted in the ICU are IL-2, IL-7, GCSF, IP10, MCP1, MIP1A, and TNF-α [88,100], while levels of IL1RA, IL8, IL9, basic FGF, GM-CSF, IFNγ, MIP1B, PDGF, and VEGF are elevated in infected than in healthy individuals [100]. IL-16, which is also found overexpressed, characterizes the initiation of humoral response, promoting CD4+ T cells and circulating DCs’ migration into lymphoid organs and is considered to be a characteristic of long-term recovery PBs [62].

## 7. Discussion

It has been almost two years since the Severe Acute Respiratory Syndrome Coronavirus (SARS-CoV-2) emerged and was designated as the causative agent of COVID-19, an infection which presents with great clinical variability, resulting in a great variety of distinctive features and extensive disease distribution, with extrapulmonary manifestations. Active research is necessary to identify all serological, clinical, epidemiological features of COVID-19, and characterize the immune response via transcriptome analysis. The identification of a number of hematological and immunological markers, as well as a panel of genes that have potential as biomarkers associated with disease progression from mild/moderate to severe/critical, will ensure the successful stratification of patients and predict immune responses, enabling effective management.

As we can conclude from the study of a great number of published papers about COVID-19, hematological parameters, cell populations subsets and transcriptomic signatures could be used as biomarkers of disease severity of COVID-19. In particular, higher levels of CRP, PCT, LDH, and liver function indices are associated with severe disease and fatal outcome. In addition, concerning cell populations, elevated levels of immature monocytes, megakaryocytes, and B cells, and decreased levels of dendritic cells (mainly CD1+ conventional dendritic cells) and T cells (especially CD3+, CD4+ and CD8+ T cells) are mainly observed in severe COVID-19 patients and as a result could be considered as markers of severe disease [7,39,40,49,50,53,54,56,67,69]. Furthermore, a decreased number of natural killer cells is correlated with severe and critical disease progression [43,49,53]. The differences in cell populations are also reflected in the differentially expressed genes between severely and mildly ill COVID-19 patients. Specifically, the upregulation of genes playing a role in B cell activation and differentiation to PBs is observed in patients with severe COVID-19 [62]. Another example is the increased expression of genes related to recruitment of monocytes and macrophages in severe patients, such as IL-6, the overproduction of which is particularly noted in critical disease [90,99]. As far as transcriptomic signatures are concerned, in severely ill COVID-19 patients, TNF-α signaling pathways are enriched, resulting in increased expression of multiple ISGs, revealing more robust activation of innate immunity in this group of patients [84], whereas in mild patients, pathways linked to IFN-α/γ responses are significantly enriched. IFN-I and II response is especially activated in moderate patients compared to mild, severe, and critical ones, and in patients who have higher viral load and prolonged viral clearance [40,69,84,95]. Besides, inflammatory mediators, such as IL-2, TNF-α, and IL-6 have been found to be linked with severe disease [98]. Finally, apoptosis-relates genes are overproduced in more clinically unstable patients [97]. In summary, the dynamic changes in biomarkers levels can be valuable tools in the prediction of the course of COVID-19 and therefore studies with larger cohorts should be established (organized) to further confirm the predictive clinical value of the current findings.

## Figures and Tables

**Table 1:** Alterations in blood-based biomarkers among patients with mild and severe COVID-19.

Blood Biomarkers	Elevated in Severe Compared to Mild COVID-19	Reduced in Severe Compared to Mild COVID-19
Hematological/Flow cytometry	neutrophil count	lymphocytes
	NLR	
	WBCs	
Inflammation	CPR, ESR. Ferritin, Cytokines (IFN-γ, TNF-α), interleukins (IL-6, IL-10)	
Thromboinflammatory	Troponin	
Coagulation	D-dimer, prothrombin time	platelets, fibrinogen
Hepatic	albumin, liver enzymes (ALT, AST, LDH)	
Muscle damage	CPK	

## Data Availability

Not applicable.

## Supplementary Materials

The following supporting information can be downloaded at: https://www.mdpi.com/article/10.3390/pathogens11030311/s1, Table S1: List of symbols, corresponding Entrez ID, names and aliases of genes differentially expressed in mild, moderate, severe and critical patients during the immune response genes in COVID-19.
