# Supplementary material for: Molecular and Clinical Prognostic Biomarkers of COVID-19 Severity and Persistence
Source: Pathogens. 2022 Mar 2;11(3):311. doi: 10.3390/pathogens11030311 (PMC8948624; doi:10.3390/pathogens11030311)
Supplement: Supplementary file 1 [file pathogens-11-00311-s001.zip › pathogens-1603156-supplementary.pdf]

pathogens

MDPI

Supplementary Material

Supplementary Table S1. List of symbols, corresponding Entrez ID, names and aliases of genes differentially expressed in mild, moderate, severe and critical patients during the immune response genes in COVID-19.

Gene Symbol	NCBI Entrez Gene	Gene Full Name	Aliases	References
ARG1	383	arginase 1	-	[39]
CCL2	6347	C-C motif chemokine ligand 2	GDCF-2, HC11, HSMCR30, MCAF, MCP-1, MCP1, SCYA2, SMC-CF	[90]
CCL3	6348	C-C motif chemokine ligand 3	G0S19-1, LD78ALPHA, MIP-1-alpha, MIP1A, SCYA3	[68,85]
CCL4	6351	C-C motif chemokine ligand 4	ACT2, AT744.1, G-26, HC21, LAG-1, LAG1, MIP-1-beta, MIP1B, MIP1B1, SCYA2, SCYA4	[68,85]
CCL5	6352	C-C motif chemokine ligand 5	D17S136E, RANTES, SCYA5, SIS-delta, SISd, TCP228, eoCP	[51]
CCL8	6355	C-C motif chemokine ligand 8	HC14, MCP-2, MCP2, SCYA10, SCYA8	[90]
CCL22	6367	C-C motif chemokine ligand 22	A-152E5.1, ABCD-1, DC/B-CK, MDC, SCYA22, STCP-1	[91]
CD2	914	CD2 molecule	LFA-2, SRBC, T11	[39]
CD4	920	CD4 molecule	CD4mut, IMD79, OKT4D	[39]
CD38	952	CD38 molecule	ADPRC 1, ADPRC1	[62]
CD44	960	CD44 molecule (Indian blood group)	CDW44, CSPG8, ECMR-III, HCELL, HUTCH-I, IN, LHR, MC56, MDU2, MDU3, MIC4, Pgp1	[68]
CD74	972	CD74 molecule	DHLAG, HLADG, II, Ia-GAMMA, p33	[68]
CD177	57126	CD177 molecule	HNA-2a, HNA2A, NB1, NB1 GP, PRV-1, PRV1	[39]
CD244	51744	CD244 molecule	2B4, NAIL, NKR2B4, Nmrk, SLAMF4	[68]
CD247	919	CD247 molecule	CD3-ZETA, CD3H, CD3Q, CD3Z, IMD25, T3Z, TCRZ	[39,51]
CX3CR1	1524	C-X3-C motif chemokine receptor 1	CCRL1, CMKBRL1, CMKDR1, GPR13, GPRV28, V28	[39]
CXCL8	3576	C-X-C motif chemokine ligand 8	GCP-1, GCP1, IL8, LECT, LUCT, LYNAP, MDNCF, MONAP, NAF, NAP-1, NAP1, SCYB8	[91]
CXCL9	4283	C-X-C motif chemokine ligand 9	CMK, Humig, MIG, SCYB9, crg-10	[91]

CXCL12	6387	C-X-C motif chemokine ligand 12	IRH, PBSF, SCYB12, SDF1, TLSF, TPAR1	[91
ELANE	1991	elastase, neutrophil expressed	ELA2, GE, HLE, HNE, NE, PMN-E, SCN1	[39]
FUT4	2526	fucosyltransferase 4	LeX, CD15, ELFT, FCT3A, FUTIV, SSEA-1, FUC-TIV	[39]
GZMK	3003	granzyme K	TRYP2	[51]
H3F3B	3021	H3 histone family member 3B	H3-3A, H3.3B, H3F3B	[88]
HMGB2	3148	high mobility group box 2	HMG2	[51]
IFI6	2537	interferon alpha inducible protein 6	6-16, FAM14C, G1P3, IFI-6-16, IFI616	[62]
IFI27	3429	interferon alpha inducible protein 27	FAM14D, ISG12, ISG12A, P27	[62]
IFIH1	64135	interferon induced with helicase C domain 1	AGS7, Hlcd, IDDM19, MDA-5, MDA5, RLR-2, SGMRT1	[91]
IFITM1	8519	interferon induced transmembrane protein 1	9-27, CD225, DSPA2a, IFI17, LEU13	[41,50,88,62]
IFITM2	10581	interferon induced transmembrane protein 2	1-8D, DSPA2c	[50,88,62]
IFITM3	10410	interferon induced transmembrane protein 3	1-8U, DSPA2b, IP15	[62]
IFNG	3458	interferon gamma	IFG, IFI, IMD69	[88]
IL-1B	3553	interleukin 1 beta	IL-1, IL1F2, IL1-BETA, IL1beta	[88]
IL-7R	3575	interleukin 7 receptor	CD127, CDW127, IL-7R-alpha, IL7RA, ILRA, CD127	[39]
IL-10	3586	Interleukin 10	CSIF, TGIF, GVHDS, CSIF IL10A	[99,91]
IRF-1	3659	interferon regulatory factor 1	MAR	[96,96]
IRF-4	3662	interferon regulatory factor 4	LSIRF, MUM1, NF-EM5, SHEP8	[51]
IRF-8	3394	interferon regulatory factor 8	H-ICSBP, ICSBP1, IMD32A, IMD32B, ICSBP	[68]
IRF-9	10379	interferon regulatory factor 9	ISGF3, ISGF3G, p48	[96]
ISG20	3669	interferon stimulated exonuclease gene 20	CD25, HEM45	[50]
ITGA2B	3674	integrin subunit alpha 2b	BDPLT16, BDPLT2, CD41, CD41B, GP2B, GPIIb, GT, GT1, GTA, HPA3, PPP1R93	[62]
KLF6	1316	Kruppel like factor 6	BCD1, CBA1, COPEB, CPBP, GBF, PAC1, ST12, ZF9	[88]

MKI67	4288	marker of proliferation Ki-67	KIA, MIB-, MIB-1, PPP1R105	[51]
MMP8	4317	matrix metallopeptidase 8	CLG1, HNC, MMP-8, PMNL-CL	[39]
MMP9	4318	matrix metallopeptidase 9	CLG4B, GELB, MANDP2, MMP-9	[39,84]
MPO	4353	myeloperoxidase	-	[39]
MSR1	4481	macrophage scavenger receptor 1	CD204, SCARA1, SR-A, SR-AI, SR-AII, SR-AIII, SRA, phSR1, phSR2	[39]
NLRC4	58484	NLR family CARD domain containing 4	AIFEC, CARD12, CLAN, CLAN1, CLANA, CLANB, CLANC, CLAND, CLR2.1, FCAS4, IPAF	[39]
ODC1	4953	ornithine decarboxylase 1	BABS, NEDBA, NEDBIA, ODC	[62]
OLFM4	10562	olfactomedin 4	GC1, GW112, OLM4, OlfD, UNQ362, bA209J19.1, hGC-1, hOLfD	[39]
PADI4	23569	peptidyl arginine deiminase 4	PAD, PAD4, PADI5, PDI4, PDI5	[39]
PAF1	54623	PAF1 homolog Paf1/RNA polymerase II complex component	F23149_1, PD2	[88]
PIM2	11040	Pim-2 proto-oncogene, serine/threonine kinase	-	[62]
PKM	5315	pyruvate kinase, muscle	CTHBP, HEL-S-30, OIP3, PK3, PKM2, TCB, THBP1, p58	[62]
POU2AF1	5450	POU class 2 associating factor 1	BOB1, OBF-1, OBF1, OCAB	[51]
PRDM1	639	PR/SET domain 1	BLIMP1, PRDI-BF1	[62]
PRL	5617	prolactin	GHA1	[88]
RETN	56729	resistin	ADSF, FIZZ3, RETN1, RSTN, XCP1	[39]
S100A8	6279	S100 calcium binding protein A8	60B8AG, CAGA, CFAG, CGLA, CP-10, L1Ag, MA387, MIF, MRP8, NIF, P8	[39,88]
S100A9	6280	S100 calcium binding protein A9	60B8AG, CAGB, CFAG, CGLB, L1AG, LIAG, MAC387, MIF, MRP14, NIF, P14	[39]
S100A12	6283	S100 calcium binding protein A12	CAAF1, CAGC, CGRP, ENRAGE, MRP-6, MRP6, p6	[39]

SAT1	6303	spermidine/spermine N1-acetyltransferase 1	DC21, KFSD, KFSDX, SAT, SSAT, SSAT-1	[88]
SELL	6402	selectin L	CD62L, LAM1, LECAM1, LEU8, LNHR, LSEL, LYAM1, PLNHR, TQ1	[51]
SLC1A4	6509	solute carrier family 1 member 4	ASCT1, SATT, SPATCCM	[62]
SPP1	6696	secreted phosphoprotein 1	BNSP, BSPI, ETA-1, OPN	[88]
STMN1	3925	stathmin 1	C1orf215, LAP18, Lag, OP18, PP17, PP19, PR22, SMN	[51]
TBX21	30009	T-box transcription factor 21	TBET, T-PET, T-bet, TBLYM	[39]
TGFB1	7040	transforming growth factor beta 1	CED, DPD1, IBDIMDE, LAP, TGF-beta1, TGFB, TGFbeta	[62]
TGM2	7052	transglutaminase 2	TGC, tTG, G(h), hTG2, TG(C)	[88]
TLR9	54106	toll like receptor 9	CD289	[88]
TRAC	28755	T-cell receptor alpha constant	IMD7, TCRA, TRA, TRCA	[39]
TPRBC1	28639	T cell receptor beta constant 1	BV05S1J2.2, TCRB, TCRBC1	[39]
XBP1	7494	X-box binding protein 1	TREB-5, TREB5, XBP-1, XBP2	[62]
